# Emerging Qualitative Research Trends (2010–2021) on Sedentary Behaviour among Older Adults: A Systematic Literature Review Protocol

**DOI:** 10.3390/ijerph182111548

**Published:** 2021-11-03

**Authors:** André Ramalho, João Serrano, Rui Paulo, Pedro Duarte-Mendes, António Rosado, João Petrica

**Affiliations:** 1 Department of Sports and Well-Being, Polytechnic Institute of Castelo Branco, 6000-266 Castelo Branco, Portugal; j.serrano@ipcb.pt (J.S.); ruipaulo@ipcb.pt (R.P.); pedromendes@ipcb.pt (P.D.-M.); j.petrica@ipcb.pt (J.P.); 2 Sport, Health & Exercise Research Unit (SHERU), Polytechnic Institute of Castelo Branco, 6000-266 Castelo Branco, Portugal; 3 Faculty of Human Kinetics, University of Lisbon, 1499-002 Cruz Quebrada, Portugal; arosado@fmh.ulisboa.pt

**Keywords:** research synopsis, qualitative study, sedentary lifestyle, ageing, geriatric, research methodology

## Abstract

In recent years, research on sedentary behaviour has increased. In this regard, there is a need for theoretical reviews that allow us to determine the past, analyse the present, and prepare the future of research in this field. The purpose of this review paper was to analyse and organise the emerging qualitative research trends (2010–2021) on the sedentary behaviour of older adults. A systematic literature search strategy was developed in various electronic scientific databases (e.g., PubMed, Web of Science, ScienceDirect, Scielo, and Scopus). The included studies were required to have different qualitative methodological approaches in terms of data collection and methods of data analysis. Studies conducted in any country and published in a peer-reviewed journal in English, Spanish, and Portuguese were considered. A thematic analysis approach was used for data extraction and synthesis, and confidence in the results was assessed using the GRADE-CERQual approach. This study may enable accurate guidelines to be established for future primary qualitative research related to sedentary behaviour.

## 1. Introduction

Based on the increasing number of publications in various scientific databases, it can be seen that the importance of generating scientific knowledge is increasing every day [1]. Therefore, in order to keep the various areas of research up to date, it is necessary to conduct literature review studies to reflect progress in a particular area. In sports science, professional development largely depends on research findings, educational programs, and the applicability of research findings in practice [2]. However, the process of professional development depends on the breadth and depth of knowledge produced about a particular research topic. As scientific data are scattered in various publication formats (e.g., articles, books, manuals, dissertations, and theses), it can be difficult for researchers and practitioners to stay up to date with the constant developments in various scientific databases. Systematic reviews make it possible to collect all relevant scientific evidence, to limit studies to different scientific fields and research topics, and to determine the reality and context of each country. This process makes it possible to determine the specific problems of a research topic in the local and international contexts, which facilitates comparative studies between countries [3].

It is scientifically proven that sedentary behaviours (SB) constitute a large part of the daily routine of the elderly population (>8 h a day) [4,5]. SB is any waking behaviour characterised by energy expenditure ≤ 1.5 metabolic equivalents (METs) while sitting, lying, or standing [6]. High levels of daily SB are associated with significant health consequences for the adult population, including the elderly [7]. The negative biopsychosocial health consequences of SB are independent of an individual’s level of physical activity [8]. However, certain sedentary activities appear to have a positive impact on psychosocial health [9,10]. In addition, it appears that computer use and reading may be associated with cognitive function in the elderly [11].

In fact, in recent years, the amount of research on SB has increased exponentially [12]. The literature reviews published on SB in the older population have followed the evidence phases for SB science guided by the Behavioural Epidemiology Framework [13]. Therefore, it is possible to identify studies that link SB to health outcomes [14,15,16,17]. In addition, other literature reviews have aimed to understand the measurement of SB and describe the prevalence and variations of SB in older adults [4,18,19,20]. In another direction, reviews have been published to identify the determinants of SB [21,22]. Finally, there are literature reviews that focus on the development and testing of interventions to reduce daily SB [23,24,25].

In March 2020, several countries banned various activities during the COVID-19 pandemic, commonly referred to as “lockdowns”. These lockdowns have the potential to impact associated levels of physical activity and SB. Along these lines, other review studies have aimed to analyze the published scientific literature on changes in physical activity and SB behaviour during and before the lockdown imposed by the COVID-19 pandemic [26,27]. In relation to sedentary behaviour, this review showed that levels of sedentary behaviour increased. Therefore, public health strategies should include the promotion of physical activity and guidance to reduce SB during the period of delivery, especially in special populations. Analysis of the impact of the COVID-19 pandemic on SB in the elderly could be an important area of future research. In terms of qualitative research, it may be important for studies to investigate the reasons that people show changes in their physical activity and/or SB.

As for the review studies summarising the qualitative evidence on SB, the review by Compernolle [28] is worth highlighting. The aim of this study was to summarise the studies on older adults’ perceptions of (a) the concept of SB, (b) the barriers and facilitators of SB, and (c) solutions and strategies to reduce SB. This review was found to be relevant to the development of recommendations that may reduce SB in older people. However, only studies published in English were selected, and studies from high-income countries were included. In addition, primary studies with participants from developing countries were not pooled. Previous literature reviews examining SB in older people have focused on analysing studies published in the most representative databases (e.g., Web of Science, Scopus, and PubMed) and papers written in English. Few reviews have analysed qualitative studies on SB published in other languages, such as Portuguese and Spanish. Moreover, for a meaningful extension of the literature, it is necessary to search for studies in several indexed databases.

In defining a research problem, researchers need to ensure that they have a good understanding of the underlying literature as it relates to the identified research problem. In this respect, there is a need for review papers that report on the progress of a particular scientific field. Therefore, researchers should not move to the next phase of research until they have clearly defined the problem at hand and have a complete understanding of the related literature [29]. The study of SB in the elderly population is important research [7,11] and requires theoretical reviews that allow us to determine the past, analyse the present, and prepare the future of research in this area. In this sense, it is imperative to carry out a systematic literature review that will allow a comprehensive analysis of the scientific production of qualitative studies on SB in the elderly population. An analysis of the scientific evidence allows us to identify the reality of a particular field of study, its evolution, and the prevailing methodological approaches [30]. Therefore, determining the reality of qualitative studies on SB in the elderly population can help identify future lines of research. The systematic literature review provides a remarkable methodological robustness [31] and the opportunity to collect all empirical evidence that meets the pre-established selection criteria [32]. Thus, the aim of this systematic literature review is to answer the following question: what are the emerging research trends in qualitative studies of SB in the older population between 2010 and 2021?

## 2. Methods

This systematic literature review protocol was developed based on the framework proposed in Preferred Reporting Items for Systematic Reviews and Meta-analysis Protocols (PRISMA-P) [33].

### 2.1. Eligibility Criteria

In this protocol of systematic synthesis of qualitative evidence, the criteria used as a reference for inclusion and exclusion of primary studies were guided by the acronym SPIDER (Sample, Phenomenon of Interest, Design, Evaluation, Research type) [34].

Primary studies that use a variety of qualitative methodological approaches (e.g., ethnography, phenomenology, life histories, grounded theory, case studies, and descriptive qualitative studies) were included in the review. In addition, studies using qualitative methods of data collection (e.g., unstructured interviews, semi-structured interviews, structured interviews, focus groups, and direct observation) and various qualitative approaches to data analysis (e.g., thematic analysis, interpretive phenomenological analysis, and content analysis) were considered. Primary studies in which data were collected using qualitative methods but which did not include qualitative analysis were not included (e.g., surveys in which responses were analysed using descriptive statistical methods). Mixed methods studies and studies published in conference proceedings, scientific papers, book chapters, and unpublished manuscripts were also not included in the evidence synthesis.

The synthesis also included primary studies that focused on the perceptions of (1) older men and women aged 65 years or older; (2) older people living in their own homes and older people living in nursing homes and residences; (3) older people with multiple comorbidities and older people without associated pathologies; (4) older people who do or do not participate in exercise programmes guided by exercise professionals, and older people who do or do not regularly engage in unguided physical activity. Studies that exclusively examined clinical populations (e.g., older adults undergoing cardiac rehabilitation) were not included in the synthesis. Studies conducted in any country and published in English, Spanish, and Portuguese in peer-reviewed journals between 2010 and 2021 were considered for the final synthesis.

### 2.2. Literature Search Strategy

A systematic literature search strategy was developed in the following electronic scientific databases: PubMed, Web of Science, ScienceDirect, Scielo, and Scopus. The aforementioned databases were selected because they revealed the previous existence of potentially critical studies related to the research question of the synthesis based on preliminary investigations and explorations. The search for the primary studies was conducted using keywords associated with the following groups of search terms: (a) SB and related terms (e.g., sedentary lifestyle, SB, prolonged sitting, sitting time, reclining time, computer time, Internet time, television, screen time, reading time, computer play, and transportation time); (b) qualitative research designs and analyses (e.g., ethnography, phenomenology, life stories, grounded theory, case studies, focus groups, descriptive qualitative studies, qualitative analysis, thematic analysis, content analysis, and interpretive phenomenological analysis); (c) participant characteristics (e.g., ageing, senior, older adult, elderly, and geriatric). The different keywords within each group were combined with a Boolean “OR”. In addition, the keywords were combined with a Boolean “AND”.

The search strategy for the PubMed scientific database (Table 1) was used as the basis for the literature search in the various databases listed. Reference lists of primary studies selected for synthesis were then reviewed to identify new studies that may have met the defined eligibility criteria. In addition, primary studies potentially relevant to the synthesis were also searched in Google Scholar and the Sedentary Behaviour Research Network database (www.sedentarybehaviour.org, accessed on 6 September 2021). Contact with experts in SB was considered to identify additional unpublished studies. These contacts could be made through the Sedentary Behaviour Research Network (www.sedentarybehaviour.org, accessed on 6 September 2021), the International Society for Physical Activity and Health (www.ispah.org, accessed on 6 September 2021), and other international research networks.

### 2.3. Study Selection and Data Extraction

The titles and abstracts of primary studies identified using the literature search strategy described above were exported to End-Note software (Clarivate, San Francisco, CA, USA). Studies that may have had duplicates were removed using the “remove duplicates” function. Then, two co-authors of the systematic review independently assessed the titles and abstracts of the studies to decide whether to include or exclude them according to the predefined eligibility criteria. At this stage, disagreements between the co-authors on the inclusion or exclusion of studies in the evidence synthesis were resolved by consensus. Subsequently, the full texts of the studies selected in the previous phase were independently analysed by two co-authors. This analysis verified whether the primary studies were included or excluded according to the predefined eligibility criteria. Disagreements between the co-authors on the inclusion or exclusion of studies in the final synthesis were resolved by consensus or, if necessary, by involving a third co-author. In addition, the authors of the primary studies were sometimes contacted to obtain additional information that may have been helpful in determining the suitability of the studies for the evidence synthesis. The process of study selection is illustrated using the PRISMA flowchart [32].

The studies included in the evidence synthesis were extracted using a data extraction sheet adapted from the Cochrane Consumers and Communication Review Group data extraction template. Thus, the following information was extracted: study title, study abstract, authors and year of study publication, country in which the study was conducted, research design used, study objectives/research questions, participant characteristics, qualitative data collection and analysis methods, qualitative results, participant citations from primary studies, implications of study findings, relevant tables, figures or images, and conclusions. In addition, data extraction included SB measurement methods when studies included in the synthesis were available. To minimise the risk of bias, data were extracted by two independent co-authors, and discrepancies were resolved by consensus. Subsequently, the extracted data are presented in a table entitled “Characteristics of included studies”.

### 2.4. Assessment of the Risk of Bias of the Studies Included in the Synthesis

The Critical Appraisal Skills Programme (CASP) checklist for assessing studies that use qualitative methods for data collection and analysis was used to assess the risk of bias of studies included in the synthesis. The assessment was based on the following criteria: study aims, methodological appropriateness, recruitment of participants, data collection procedures, data analysis, reflexivity, ethical considerations, conclusions, and research contribution. In addition, the text boxes listed under each criterion were used to document explanations of the ratings for future review. Based on all the responses to the questions and their explanations, the studies were rated as: “Minor Concern” if they met at least 8 of the 10 criteria; “Moderate Concern” if they met between 5 and 7 of the criteria; and “Serious Concern” if they met 4 or fewer of the criteria [28]. Therefore, two co-authors independently assessed the studies included in the qualitative evidence synthesis against the listed items. Disagreements between the co-authors on the assessment of risk of bias in the synthesis were resolved by consensus or, if necessary, by involving a third co-author. The critical appraisal was part of the assessment of the co-authors’ confidence in the risk of bias for each primary study included in the synthesis. Thus, this assessment did not serve as the basis for excluding a study from the final evidence synthesis.

### 2.5. Data Analysis

The studies that were integrated into the final synthesis were subjected to qualitative analysis. In this sense, a thematic analysis [35] was conducted from the data extraction sheet used. The data were imported into the MAXQDA software (VERBI Software, Berlin, Germany). The first step consisted of familiarising ourselves with the data, reading it thoroughly, and noting the first ideas that emerged from the data. The second phase was to define initial codes, systematically code the data, and group the relevant data for each code. In the third phase, a more comprehensive analysis was conducted, grouping the initial codes into potential themes, and bringing together all the relevant data for each theme. In the fourth phase, the potential themes were matched with the previously coded extracts to generate individual unique themes. In the fifth stage, the themes were reviewed again to refine their specifics by developing clear titles for each theme. In this way, the themes were developed to ensure their uniqueness and completeness. Finally, in the sixth stage, the themes were written down in an analytical report.

In the first phase, two co-authors independently developed and presented a thematic analysis according to the procedures described above. Regular meetings were then held between all authors of the study to discuss and interpret themes emerging from the data. At this stage, disagreements between co-authors were resolved by consensus. This form of discussion can ensure that the themes reflect the full range of data reviewed [36]. Thus, this approach allowed for a scientific synthesis of the findings of a qualitative design and a new understanding of the scientific production of qualitative data related to the study of SB in the elderly population.

### 2.6. Assessment of Confidence in the Synthesis Findings

We used Confidence in the Evidence from Reviews of Qualitative Research (GRADE-CERQual) to assess co-authors’ confidence in the results of evidence synthesis [37,38]. This approach, based on the principles of GRADE evaluation [39] and assessing the risk of study bias [40], is currently the standard procedure for assessing confidence in the conclusions of qualitative evidence syntheses [31]. The CERQual approach can be used to assess the following parameters [37]: (1) methodological limitations of included studies; (2) coherence of the review outcome; (3) adequacy of data contributing to a review outcome; (4) relevance of included studies to the review question. The above parameters can be used to assess the degree of confidence in each outcome of the synthesis. In this sense, the results of the evidence synthesis can be classified as follows: high confidence level; moderate confidence level; low confidence level; very low confidence level. The results of the CERQual assessments are presented in a table entitled “Summary of qualitative results”. This table includes the confidence level for each evidence synthesis result and the rationale for the assessments performed, based on the GRADE-CERQual approach [38].

## 3. Conclusions

In recent years, research on SB has increased. In this regard, there is a need for theoretical reviews that allow us to determine the past, analyse the present, and prepare for the future of research in this field. This evidence synthesis focused on systematising the emerging qualitative research trends (2010–2021) on the study of SB in the elderly population. In this way, the review incorporated those studies that have extensively examined the perceptions of older people from different countries regarding excessive daily SB. When defining a new research problem, it is important to be fully aware of the literature on the area to which the researcher intends to contribute. Therefore, based on this study, it might be possible to identify the real and concrete problems related to qualitative scientific production on the study of SB in the elderly population. In this sense, future research should be based on the problems of the phenomenon under study rather than on theoretical and methodological constraints. Moreover, this review allowed us to determine the methodological strengths and weaknesses of the studies included in the synthesis. Through a better understanding of these dimensions, it will be possible to present an accurate and relevant set of future qualitative lines of research on SB in older people.

## Figures and Tables

**Table 1:** PubMed search strategy.

1. Sedentary lifestyle [MeSH Terms] 2. (((sedentary behavior [tiab]) OR sedentary behaviour [tiab]) OR sedentary lifestyle [tiab]) 3. prolonged sitting [tiab] 4. computer [MeSH Terms] 5. ((computer use [tiab]) OR computer usage [tiab]) OR computer time [tiab] 6. television [MeSH Terms] 7. ((television viewing [tiab]) OR television watching [tiab]) OR television time [tiab] 8. ((TV viewing [tiab]) OR TV watching [tiab]) OR TV time [tiab] 9. ((screen watching [tiab]) OR screen use [tiab]) OR screen time [tiab] 10. ((screen entertainment [tiab]) OR screen behaviour [tiab]) OR screen behavior [tiab] 11. reading time [tiab] 12. automobile driving [MeSH Terms] 13. transport time [tiab]
AND
14. qualitative research [MeSH Terms] 15. ethnography [tiab] 16. phenomenology [tiab] 17. life stories [tiab] 18. grounded theory [tiab] 19. case studies [tiab] 20. focus groups [tiab] 21. descriptive qualitative study [tiab] 22. qualitative investigation [tiab] 23. qualitative analysis [MeSH Terms] 24. thematic analysis [tiab] 25. content analysis [tiab] 26. interpretive phenomenological analysis [tiab] 27. thematic synthesis [tiab] 28. narrative analysis [tiab] 29. interviews [MeSH Terms] 30. in-depth interviews [MeSH Terms] 31. structured interviews [tiab] 32. semi-structured interviews [tiab] 33. unstructured interviews [tiab] 34. ((observation [tiab]) OR participant observation [tiab]
AND
35. aging [MeSH Terms] 36. ((senior [tiab]) OR senior citizens [tiab] 37. older adult* [tiab] 38. elderly [tiab] 39. older people [tiab] 40. geriatric OR geriatrics

## Data Availability

Not applicable.
